# Alterations in White Matter Integrity in Young Adults with Smartphone Dependence

**DOI:** 10.3389/fnhum.2017.00532

**Published:** 2017-11-02

**Authors:** Yuanming Hu, Xiaojing Long, Hanqing Lyu, Yangyang Zhou, Jianxiang Chen

**Affiliations:** ^1^Department of Radiology, Shenzhen Traditional Chinese Medicine Hospital, Shenzhen, China; ^2^Paul C. Lauterbur Research Center for Biomedical Imaging, Shenzhen Institutes of Advanced Technology, Chinese Academy of Sciences, Shenzhen, China; ^3^Department of Radiology, Shenzhen Children’s Hospital, Shenzhen, China

**Keywords:** smartphone dependence, diffusion tensor imaging, tract-based spatial statistics, white matter integrity, behavioral impairment

## Abstract

Smartphone dependence (SPD) is increasingly regarded as a psychological problem, however, the underlying neural substrates of SPD is still not clear. High resolution magnetic resonance imaging provides a useful tool to help understand and manage the disorder. In this study, a tract-based spatial statistics (TBSS) analysis on diffusion tensor imaging (DTI) was used to measure white matter integrity in young adults with SPD. A total of 49 subjects were recruited and categorized into SPD and control group based on their clinical behavioral tests. To localize regions with abnormal white matter integrity in SPD, the voxel-wise analysis of fractional anisotropy (FA) and mean diffusivity (MD) on the whole brain was performed by TBSS. The correlation between the quantitative variables of brain structures and the behavior measures were performed. Our result demonstrated that SPD had significantly lower white matter integrity than controls in superior longitudinal fasciculus (SLF), superior corona radiata (SCR), internal capsule, external capsule, sagittal stratum, fornix/stria terminalis and midbrain structures. Correlation analysis showed that the observed abnormalities in internal capsule and stria terminalis were correlated with the severity of dependence and behavioral assessments. Our finding facilitated a primary understanding of white matter characteristics in SPD and indicated that the structural deficits might link to behavioral impairments.

## Introduction

Smartphones are becoming a vital part of our lives. These portable “mini-computers” help keep us connected, organized and entertained anywhere and anytime (Samaha and Hawi, 2016). Smartphones allow us to make calls, check and send emails, use chat applications for texting or voice calls, manage calendars, map driving or walking routes, take/edit/store/share videos and photos, play games or music, and more (Long et al., 2016). Smartphones have been changing the way we live—how we feel, how we behave, how we communicate and how we age (Dennison et al., 2013; Sarwar and Soomro, 2013; Abu-Shanab and Haddad, 2015).

However, accompanying the popularization of smartphones, their overuse has become an epidemic around the world. In the USA, 64% of American adults owned a smartphone in 2014 according to a survey by the Pew Research Center, up from 35% in 2011. As many as 15% of American young adults between 18 years and 29 years of age were found to be heavily dependent on smartphones (Smith, 2015; Long et al., 2016). In China, the Ministry of Industry and Information Technology announced that smartphone ownership had exceeded 1.3 billion by 2016, which is 95.5% of the total population (Long et al., 2016). The prevalence of problematic smartphone use among Chinese adolescents and undergraduates was estimated to be 21.3% to 26.2% (Long et al., 2016; Tao et al., 2016). Smartphone ownership initiation also exhibits a younger tendency. In Spain, the proportions of children who own a cell phone at age 10, 12 and 14 are 30%, 70% and 83%, respectively. Furthermore, Spanish children habitually access such devices beginning from 2–3 years of age (Protegeles, 2014).

Smartphone dependence (SPD), also called smartphone addiction or problematic mobile phone use, is regarded as a behavioral addiction, as opposed to a substance addiction, such as drug or alcohol abuse. An increasing number of studies have been conducted on behavioral addictions to videogames (Fisher, 1994), food (Oxford, 2001), shopping (O’Guinn and Faber, 1989; Clark and Calleja, 2008), work (Andreassen et al., 2010), online sex (Griffiths, 2004; Young, 2008) and the Internet (Young, 2004; Beard, 2005). As a subset of addictive behaviors, SPD may share many common properties with other kinds of addiction disorders (Bianchi and Phillips, 2005; Billieux, 2012), especially Internet addiction. For example, they may present similar problematic manifestations (De-Sola Gutiérrez et al., 2016), including conscious use in dangerous situations or prohibited contexts (Billieux et al., 2008), loss of interest in other activities (Griffiths, 2000), physical and mental impairment (Chóliz, 2012), social and familial interruptions (Sansone and Sansone, 2013), and anxiety and loneliness with restricted usage (Ha et al., 2008).

Studies on Internet addiction have focused on the behaviors it involves, its influence on daily life and social performance, clinical diagnosis and treatment (Caplan, 2002; Shaw and Black, 2008; Tao et al., 2010, 2016; The Ministry of Industry and Information Technology of China, 2016), as well as effects on brain structures and functions (Yuan et al., 2011; Lin et al., 2012; Wee et al., 2014; Sepede et al., 2016). High-resolution magnetic resonance imaging has allowed enhanced sensitivity and power to detect morphological, functional, regional and network changes in the brain, which has facilitated exploration of the neural substrates of addiction. Evidence has shown altered gray matter density (Yuan et al., 2011; Weng et al., 2013), cortical thickness (Hong et al., 2012; Yuan et al., 2013), cerebral blood flow (Feng et al., 2013), and functional connectivity (Ding et al., 2013; Hong et al., 2013) in regions involved in addictive or compulsive behaviors in subjects with Internet addiction. Changes in white matter tracts (Yuan et al., 2011; Dong et al., 2012; Lin et al., 2012) were also detected by diffusion tensor imaging (DTI), a sensitive tool that reflects the water diffusion characteristics of white matter.

We hypothesized that due to its similarities to other behavioral addictions, SPD may also be associated with impairments of white matter fibers (Wang et al., 2016). In this study, we investigated white matter integrity in young adults with SPD using observer-independent tract-based spatial statistics (TBSS) analysis with DTI and linked the quantitative variables of brain structures to the behavioral measures of SPD.

## Materials and Methods

### Subjects

This study was approved by the institutional review board of Shenzhen Traditional Chinese Medicine Hospital. The purpose and procedures of the study was detailedly introduced to volunteers. Informed written consent was obtained from each subject upon participating the study. Forty-nine young adult volunteers were recruited from the local community. No significant difference was found in handedness or in social and educational background among subjects. The medical history of each subject was reviewed carefully to exclude those with endocrinal, neurological, or psychiatric disorders. Subjects who had ever taken benzodiazepines or antipsychotic drugs were also excluded. The demographic information is summarized in Table 1.

**Table 1:** Demographic information and questionnaire scores of subjects.

Groups	Control (*n* = 24)	SPD (*n* = 25)	*P* value
Age	23.07 ± 2.01	22.11 ± 1.78	0.1684
Gender (M/F)	12/12	14/11	0.4335
Education (years)	15.11 ± 1.53	14.72 ± 0.75	0.3450
Phone usage time per week	25.2 ± 1.27	41.9 ± 3.65	<0.01
MPATS	25.71 ± 7.83	62.4 ± 6.79	<0.01
BIS	32.45 ± 10.47	50.13 ± 15.31	<0.01

### Group Categorization

Qualified subjects were categorized into the control group or SPD group according to their score on the Mobile Phone Addiction Tendency Scale (MPATS; Xiong et al., 2012). The MPATS questionnaire utilizes the criteria for Internet addiction defined by Young (1998) as a reference, with terminology adapted to mobile phone use. It consists of 16 items including questions such as “Do you check your mobile phone many times a day even when your phone doesn’t ring, beep, or buzz?” “Would you feel anxious if your cell phone or network was unreachable?” and “Have you ever put a relationship or job at risk due to excessive cell phone use?” The full list of MPATS questions can be seen in the Supplementary File. The items were rated using a five-point Likert scale (1 = not at all, 2 = rarely, 3 = occasionally, 4 = often, 5 = always). Scores higher than 50 were classified as SPD. The Cronbach’s alpha of the scale is 0.81, indicating good reliability (Xiong et al., 2012).

The Barratt Impulsiveness Scale-11 (BIS-11) was used to assess the behavioral construct of impulsiveness (Patton et al., 1995). The BIS-11 is composed of 30 items describing attentional, motor, and non-planning impulsivity behaviors and preferences. Items are scored on a four-point Likert scale (1 = rarely/never, 2 = occasionally, 3 = often, 4 = almost always/always). The reliability of the general scale is 0.80.

The usage rate of each function of a smartphone, including traditional calling and texting, chat apps, gaming and entertainment, media and Internet browsing, time checking and camera shooting, as well as sleep status, were also included in the survey questionnaire for assessment of the basic conditions.

### MRI Acquisition

MR scanning was conducted on a 3T scanner (MAGNETOM Skyra, Siemens, Erlangen, Germany) with a 32-channel head coil. Diffusion-weighted single-shot echo-planar imaging sequences with a b value of 1000 s/mm^2^ in 64 gradient directions were used with parameters of TR/TE = 4200/85 ms, FOV = 250 mm × 250 mm, image matrix = 128 × 128, in-plane resolution = 3 mm × 3 mm × 3 mm, iPAT acceleration factor = 2, number of b_0_ volumes = 8.

### Image Processing and Analysis

The final b_0_ image was calculated by averaging the eight b_0_ volumes we obtained. A standard voxel-wise TBSS analysis was performed using FSL (version 5.0.10)^1^. First, brain extraction was performed, followed by correction for head motion and eddy currents using the FSL-FDT toolbox. Then a tensor model was fit to the raw diffusion images, from which fractional anisotropy (FA) and mean diffusivity (MD) maps were computed. The group mean FA image was calculated and thinned to create a mean FA skeleton (Dennison et al., 2013). Nonlinear registration was used to align the FA maps of all subjects to the target FA image, which was chosen as the “typical image” among subjects. The warped images were then normalized to MNI space^2^. Looking for the largest FA voxel perpendicular to each point on the mean skeleton, the individual FA skeletonized image was created by projecting local FA maxima onto the group template skeleton (Smith et al., 2006). MD maps were co-registered to the MNI space and analyzed as FA maps. Whole-brain voxel-wise analyses between the control and SPD group were performed using the FSL Randomise tool^3^, which implements permutation-based inference (with 5000 permutations in this study). Multiple comparison correction was performed using the threshold-free cluster enhancement (TFCE) option to control the false positive error as 0.05.

Average FA and MD values were calculated in specific regions of interest (ROI), which were defined by the Johns Hopkins stereotaxic white matter atlas (Mori et al., 2008) and demonstrated significant differences in group analysis. The values were correlated to BIS scores while adjusting for weekly time using a smartphone and to MPATS scores within each group while regressing out BIS scores.

## Results

No significant differences in age, gender, or years of education were found between the control and SPD. Hours of phone use per week, MPATS, and BIS scores were all notably higher in the SPD group than in the control group (as shown in Table 1).

**Figure 1:**
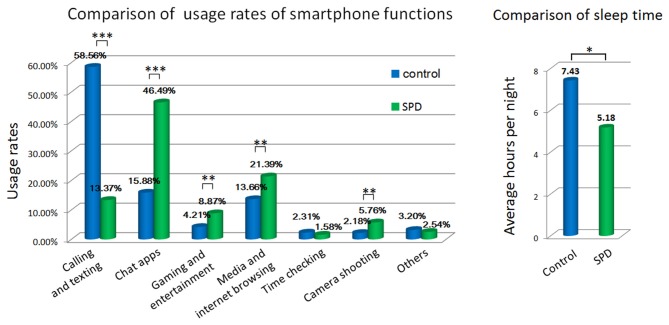
Comparison of behavioral measures by questionnaire. *p < 0.01, **p < 0.001, ***p < 0.0001.

The usage rates of calling and texting, chat apps, gaming and entertainment, media and Internet browsing, and camera shooting were significantly different in the control and SPD groups (Figure 1). SPD subjects spent much more time on chat apps, gaming and entertainment, media and Internet and camera shooting, while ordinary calling and texting remained the major purpose of smartphone usage in control subjects.

Sleeping hours also differed significantly between controls and SPD subjects. Control subjects reported an average sleep time of 7.43 h per night, whereas SPD subjects slept 5.18 h per night on average.

**Figure 2:**
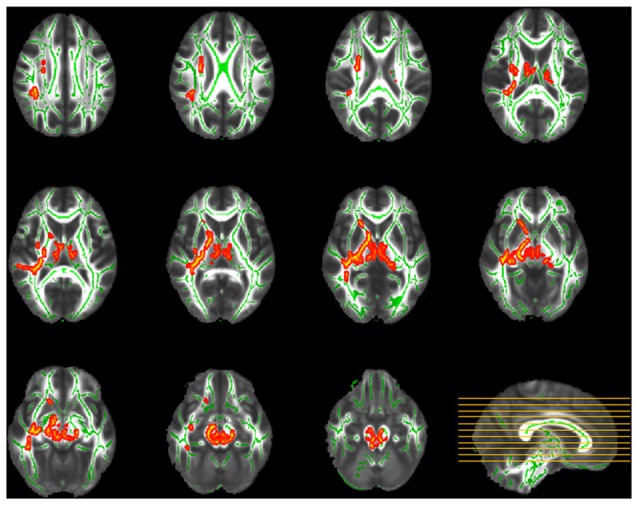
Tract-based spatial statistics (TBSS) group comparison of fractional anisotropy (FA; red–yellow: control > smartphone dependence (SPD)). Significantly lower FA was found in SPD in the highlighted areas including right superior longitudinal fasciculus (SLF), superior corona radiata (SCR), anterior limb of the internal capsule (ALIC), posterior limb of the internal capsule (PLIC), external capsule, sagittal stratum, fornix/stria terminalis, bilateral cerebral peduncle, superior and middle cerebellar peduncles, medial lemniscus and pontine crossing tract.

**Figure 3:**
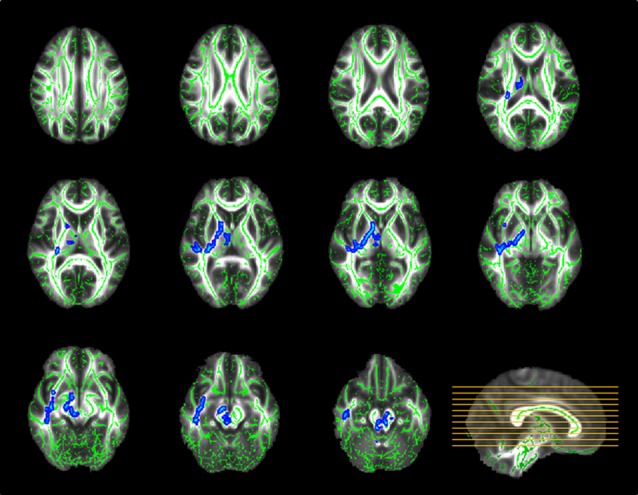
TBSS group analysis of mean diffusivity (MD; blue–light blue: control < SPD). Significantly higher MD was found in SPD relative to the control group in the highlighted areas, most of which overlapped with those with significant differences in FA.

TBSS analysis demonstrated significant between-group discrepancies in FA. Remarkably lower FA values were identified in the SPD group than in the control group, mainly in the right hemisphere, including the superior longitudinal fasciculus (SLF), superior corona radiata (SCR), anterior limb of the internal capsule (ALIC), posterior limb of the internal capsule (PLIC), external capsule, sagittal stratum and fornix/stria terminalis, as well as the bilateral cerebral peduncle, bilateral superior and middle cerebellar peduncles, bilateral medial lemniscus and pontine crossing tract (Figure 2). In contrast, in most of the aforementioned areas, significantly higher MD values were found in the SPD group than in the control group (Figure 3). No areas of higher FA or lower MD were found in the SPD group.

**Figure 4:**
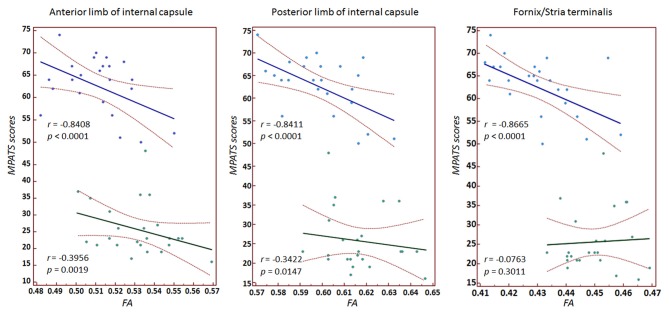
Correlation between mobile phone addiction tendency scale (MPATS) scores and FA in specific regions of interest (ROIs). A strongly negative relationship was observed between MPATS and FA in SPD.

**Figure 5:**
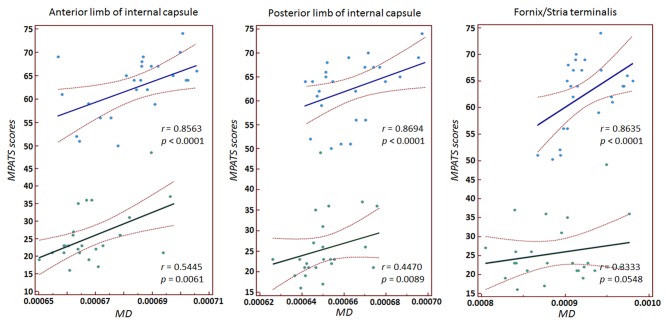
Correlation between MPATS scores and MD in specific ROIs. The results demonstrated a strongly positive relationship between MPATS and MD in SPD.

**Figure 6:**
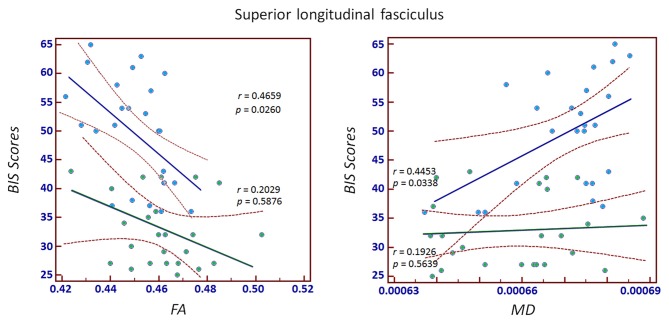
Correlation between Barratt Impulsiveness Scale (BIS) scores and FA/MD in the SLF. The results demonstrated a strongly positive correlation between BIS and FA and a negative correlation between BIS and MD.

Significant correlations between MPATS scores and mean FA and MD values were observed in the SPD group in the ALIC, PLIC and fornix/stria terminalis; these correlations were slightly weaker in the control group (Figures 4, 5). There correlation between MPATS scores and FA was significantly negative, while the correlation between MPATS and MD was positive. BIS scores were only significantly correlated with DTI measures in the SLF (Figure 6). BIS was strongly positively correlated with FA and negatively correlated with MD.

## Discussion

Cell phones are constantly being improved by expanding upon their functionalities, which in turn increases the likelihood of overuse and addiction (De-Sola Gutiérrez et al., 2016). Smartphone addiction may affect individual physical and psychological health, academic and work performance, as well as social communication (Sarwar and Soomro, 2013; Abu-Shanab and Haddad, 2015). In this study, we collected DTI to investigate white matter integrity in SPD based on whole-brain voxel-wise TBSS analysis. We also revealed the relationship between fiber abnormalities and behavioral measures of SPD.

### Influence on Sleep

The health issues that come with overuse of smartphones include text neck, wrist and back problems (Kim and Kim, 2015; Lee et al., 2015), numb fingers (İnal et al., 2015), vision loss (Kim et al., 2012, 2014; Moon et al., 2014), as well as disrupted sleep timing. In our study, SPD subjects reported significantly fewer sleep hours and worse sleep quality compared to controls, which is consistent with previous findings that mobile phone addiction was a risk factor for poor sleep quality (Sahin et al., 2013; Liu et al., 2017). This may be due not only to SPD subjects’ difficulty in resisting the temptation to use their phones before and during bedtime, but also to the sleep-disrupting sounds of alerts and effects of blue light throughout the night. The blue light from electronic devices is picked up by special cells at the back of the eyeball and leads to the suppression of melatonin, a hormone involved in sleep timing and circadian rhythms, which may push back sleep time twice as long as coffee (Burke et al., 2015).

### Influence on White Matter Microstructural Properties

In addition to perceptible changes in sleeping patterns, alterations in white matter microstructural properties were also identified in SPD subjects. The TBSS results revealed decreased FA and increased MD values in the SLF, SCR, anterior and posterior limbs of the internal capsule, external capsule, sagittal stratum, fornix/stria terminalis, cerebral peduncle, superior and middle cerebellar peduncles, medial lemniscus and pontine crossing tract.

The external capsule is a collection of white matter fibers which are thought to connect the cerebral cortex and the striatum through corticostriatal fibers (Hwang et al., 2014). Microstructural alterations in the external capsule have been found to be related to risk for cognitive dysfunction (Hwang et al., 2014), emotion regulation (Korgaonkar et al., 2011), as well as addiction and substance abuse (Upadhyay et al., 2010; Lin et al., 2012). In the present study, compared with normal subjects, individuals with SPD showed significantly lower FA in the external capsule, which is consistent with accumulating evidence suggesting that damage to the corticostriatal circuits may underlie the pathophysiology of addiction (Aston-Jones, 2015; Haber, 2016).

The internal capsule is a white matter structure situated adjacent to the ventral striatum, separating the thalamus and caudate from the putamen. Previous studies have demonstrated abnormal white matter features of the internal capsule in several types of addictions, including alcoholism (Herting et al., 2011), gambling (Yip et al., 2017), drug abuse (Wang et al., 2013; Li et al., 2016), and Internet addiction (Lin et al., 2012). Our finding that SPD involves altered directional organization and architectural integrity in the internal capsule is consistent with previous results. FA within the internal capsule has been shown to be positively correlated with reward-related ventral striatal activity in healthy adults (Koch et al., 2014), which suggests that reduced fiber anisotropy in this region may partially contribute to the structural or functional abnormalities in the ventral striatum previously reported in addictive populations (Belin and Everitt, 2008; Balodis et al., 2012; Yip et al., 2017).

The internal capsule is an important area with high concentrations of both motor and sensory projection fibers. The thalamic radiation, corticospinal and sensory tracts constitute a large part of the anterior and posterior limbs of the internal capsule. To construct the sensory fibers, the medial lemniscus ascends to the thalamus and travels in the internal capsule via third-order neurons. Then, fibers pass upward through the corona radiata to the primary sensory area of the cerebral cortex and adjacent cortical areas for further higher-level processing (Siegel and Sapru, 2011). For the corticospinal tracts, these descending fibers mainly originate from motor and sensory cortices and continue on through the corona radiata and internal capsule, then enter midbrain through the cerebral peduncle and pass through the medulla to the spinal cord (Siegel and Sapru, 2011). White matter changes in the corona radiata, internal capsule, medial lemniscus and cerebral peduncle may result in impairment of proprioception (Jang and Kwon, 2016; Cho and Lee, 2017), reduced sense of discriminative touch (Cerrato et al., 2000), or sensorimotor deficits (Kumral and Bayülkem, 2003), which have been found in smartphone addicts (Lee and Seo, 2014) and people with other dependent behaviors (Blanco-Hinojo et al., 2017; Weinstein et al., 2017).

The SLF is an association fiber tract that connects the frontal, occipital, parietal and temporal lobes (Koch et al., 2014). It is thought to be highly relevant for the processing of attention, memory, emotion and language (Yip et al., 2017). There is a growing perception that problematic use of smartphones may have a sustained negative impact on thinking, remembering, attention and emotion regulation (Wilmer et al., 2017). Changes in FA and MD in SLF indicate altered microstructural integrity, which may account for the variation in executive and emotional functioning observed in this disorder (Ward et al., 2017).

The stria terminalis is a bundle of fibers in the brain that serves as a major pathway carrying messages to and from the amygdala. Studies have shown a possible relation between the stria terminalis and addiction processes (Dagher et al., 2009; O’Daly et al., 2012). Emerging evidence suggests that the stria terminalis may play a significant role in anxiety (Avery et al., 2016). Therefore, future work is needed to investigate emotional changes and their connection to brain alterations in SPD subjects.

### Smartphone Dependence and Internet Addiction

SPD is considered one form of technological addiction, a type of behavioral addiction that involves non-chemical human–machine interaction, as defined by Griffiths (2000). A smartphone has the combined features of a mobile phone and other mobile devices, such as a media player, camera, GPS, and computer, with various applications that require Internet access. Due to the connectivity of smartphones to the Internet, SPD may result in physical, mental and psychosocial problems similar to Internet addiction (Billieux et al., 2008). Neuroimaging research on excessive Internet use has attracted increasing focus. Studies have found that problematic use of the Internet is linked to both functional and structural brain changes (Yuan et al., 2011; Dong et al., 2012; Lin et al., 2012). DTI studies demonstrated microstructural abnormalities in the prefrontal area, corpus callosum, cingulum, inferior fronto-occipital fasciculus, corona radiata, internal and external capsules and parahippocampal gyrus in Internet addiction disorder (Yuan et al., 2011; Lin et al., 2012). Along with similar behavioral symptoms to Internet addiction, SPD has been found to exhibit similar white matter changes in some regions, including the corona radiate and internal and external capsules. However, since there are differences between SPD and Internet addiction in the specific sources of addictive content and concrete applications (Ha et al., 2008), the associated brain changes may vary. For example, the abnormal changes in the corpus callosum previously found in Internet addiction were absent in SPD. Additionally, the severity of addiction may also contribute to the divergence of tissue abnormality.

### Relationship between White Matter Integrity and Behavior

In the behavioral assessment, SPD subjects showed significantly higher scores on MPATS, which represented the severity of dependence. MPATS was modified from Young (1998) diagnostic questionnaire for Internet addiction criteria and specifically adjusted for SPD evaluation. We observed a significantly negative relationship between FA and MPATS as well as a significantly positive relationship between MD and MPATS in the internal capsule and fornix/stria terminalis in SPD subjects. These results indicated that SPD subjects with higher MPATS scores were more likely to have weaker directional organization in the motor and sensory fibers connected through the internal capsule and in the afferent/efferent pathway to the amygdala connected through the stria terminalis. Moreover, the relationship between white matter structural indices and behavior measures in SPD suggests that DTI quantitative parameters can not only be used as predictors of the severity of dependence, but also clarify potential targets for the treatment of SPD.

Evidence has shown that impulsivity plays a role in the development and perpetuation of addiction (Hwang et al., 2014; Aston-Jones, 2015; Haber, 2016). High impulsivity has been identified as a risk factor for smartphone addiction (Belin and Everitt, 2008; Balodis et al., 2012). In the present study, BIS scores were significantly higher in the SPD group compared to controls, which suggested a strong link between impulsivity and smartphone addiction predisposition, consistent with existing findings. FA and MD values were also highly correlated with BIS scores when considering SPD and controls as a whole. However, for each individual group, significant correlations between structural metrics and impulsivity level were only found in SLF. SLF is linked to the relay of information across relatively long distances among the frontal, occipital, parietal and temporal lobes (Koch et al., 2014) and is thought to be involved in executive functioning and emotion regulation (Yip et al., 2017). Our results suggest that subjects with higher impulsivity, particularly SPD subjects, may have more difficulty in conducting or controlling their executive and emotional behaviors.

### Limitations

There are some limitations to this study. First, there is no comprehensive and standardized measuring scale for the diagnosis of SPD or smartphone addiction. Moreover, the assessment of SPD and the measurement of personality factors in our study relied on self-report questionnaires. More objective indices may increase the reliability of SPD categorization and severity determination. Second, the participants in this study were recruited from a relatively small community with similar educational and social backgrounds, which restricted the context of smartphone overuse, which is known to have a wide range of reasons. Third, evaluation of psychological and other factors was not comprehensively conducted in this study. Although endocrinal, neurological and psychiatric illnesses as well as substance abuse behaviors such as drug, alcohol and cigarette abuse were excluded, emotional and mental conditions such as anxiety, loneliness, impatience and obsessive-compulsive behavior that may also be linked to SPD were not considered. Finally, TBSS analysis has a fundamental limitation (Korgaonkar et al., 2011). TBSS is not a voxel-wise method but a comparison of the skeletonized projections of diffusion metrics, which may lead to problems such as losing orientation information, ambiguous assignment of voxels lying in the merging areas of different structures, and difficulty in interpreting the results (Korgaonkar et al., 2011; Tang et al., 2016). However, TBSS can still provide a preliminary delineation of how white matter differs in the target cohort and its relationship to dependent behaviors.

In conclusion, this study investigated the whole-brain white-matter microstructural characteristics affected by SPD, identifying tract integrity by conducting TBSS analysis of DTI. The results demonstrated that SPD is characterized by white matter changes in brain regions involved in motion, sensation, executive functions and emotional processing. The correlations between the quantitative variables of white matter and the behavioral measures indicated that white matter integrity in critical regions may help identify a potential treatment target for SPD. However, from the present study, we cannot infer whether SPD caused the impairments in white matter microstructures or whether the variations in brain structures predisposed individuals to SPD. Future work including longitudinal information and more detailed analysis of psychological functions is needed to clarify the development of SPD.

## Author Contributions

YH, XL and JC: conceived and designed the experiments; wrote the article; YH, JC and YZ: performed the experiments; XL, HL and JC: analyzed the data.

## Conflict of Interest Statement

The authors declare that the research was conducted in the absence of any commercial or financial relationships that could be construed as a potential conflict of interest.

## Footnotes

^1^https://fsl.fmrib.ox.ac.uk/fsl/fslwiki/TBSS

^2^http://imaging.mrc-cbu.cam.ac.uk/imaging/MniTalairach

^3^https://fsl.fmrib.ox.ac.uk/fsl/fslwiki/Randomise
