# Supplementary material for: Alterations in White Matter Integrity in Young Adults with Smartphone Dependence
Source: Front Hum Neurosci. 2017 Nov 2;11:532. doi: 10.3389/fnhum.2017.00532 (PMC5673664; doi:10.3389/fnhum.2017.00532)
Supplement: Supplementary file 1 [file Data_Sheet_1.doc]

**Supplementary File**

Mobile Phone Addiction Tendency Scale

| Number | Items | Not at all | Rarely | Occasio-  nally | Often | Always |
| --- | --- | --- | --- | --- | --- | --- |
| 1 | You checked your mobile phone many times a day even when your phone didn't ring, beep, or buzz. | 1 | 2 | 3 | 4 | 5 |
| 2 | You preferred talking with your friend or family members via mobile phone rather than face-to-face. | 1 | 2 | 3 | 4 | 5 |
| 3 | You kept calling your friend if he/she was late for appointments, otherwise you felt anxious or irritable. | 1 | 2 | 3 | 4 | 5 |
| 4 | You felt ill-at-ease or uncomfortable if you can’t use your mobile phone for a long period of time. | 1 | 2 | 3 | 4 | 5 |
| 5 | You put a relationship or job at risk due to excessive mobile phone use. | 1 | 2 | 3 | 4 | 5 |
| 6 | You felt lonely without your mobile phone. | 1 | 2 | 3 | 4 | 5 |
| 7 | You felt more confident if talking via mobile phone. | 1 | 2 | 3 | 4 | 5 |
| 8 | You tried time and again to shorten your mobile phone use but failing all the time. | 1 | 2 | 3 | 4 | 5 |
| 9 | You experienced auditory hallucinations of mobile phone sounds while not using it. | 1 | 2 | 3 | 4 | 5 |
| 10 | You felt mobile phone had enriched your life especially with a lot of calls and messages. | 1 | 2 | 3 | 4 | 5 |
| 11 | You always prepared your charging pack to keep your mobile phone from being power off. | 1 | 2 | 3 | 4 | 5 |
| 12 | You felt that not being able to use your mobile phone would be as painful as losing a friend. | 1 | 2 | 3 | 4 | 5 |
| 13 | Your friend pointed out that you used your mobile phone too much. | 1 | 2 | 3 | 4 | 5 |
| 14 | You felt anxious when your mobile phone or network was unreachable. | 1 | 2 | 3 | 4 | 5 |
| 15 | You had difficulties in concentrating to your study or work due to mobile phone use. | 1 | 2 | 3 | 4 | 5 |
| 16 | You brought your mobile phone to the toilet even when you are in a hurry to get there. | 1 | 2 | 3 | 4 | 5 |
